# Orphan G protein-coupled receptors: the ongoing search for a home

**DOI:** 10.3389/fphar.2024.1349097

**Published:** 2024-02-29

**Authors:** Amie Jobe, Ranjit Vijayan

**Affiliations:** ^1^ Department of Biology, College of Science, United Arab Emirates University, Al Ain, United Arab Emirates; ^2^ The Big Data Analytics Center, United Arab Emirates University, Al Ain, United Arab Emirates; ^3^ Zayed Bin Sultan Center for Health Sciences, United Arab Emirates University, Al Ain, United Arab Emirates

**Keywords:** GPCRs, orphan receptors, seven transmembrane receptors, 7TM receptors, GPCR deorphanization

## Abstract

G protein-coupled receptors (GPCRs) make up the largest receptor superfamily, accounting for 4% of protein-coding genes. Despite the prevalence of such transmembrane receptors, a significant number remain orphans, lacking identified endogenous ligands. Since their conception, the reverse pharmacology approach has been used to characterize such receptors. However, the multifaceted and nuanced nature of GPCR signaling poses a great challenge to their pharmacological elucidation. Considering their therapeutic relevance, the search for native orphan GPCR ligands continues. Despite limited structural input in terms of 3D crystallized structures, with advances in machine-learning approaches, there has been great progress with respect to accurate ligand prediction. Though such an approach proves valuable given that ligand scarcity is the greatest hurdle to orphan GPCR deorphanization, the future pairings of the remaining orphan GPCRs may not necessarily take a one-size-fits-all approach but should be more comprehensive in accounting for numerous nuanced possibilities to cover the full spectrum of GPCR signaling.

## 1 Introduction

**FIGURE 1:**
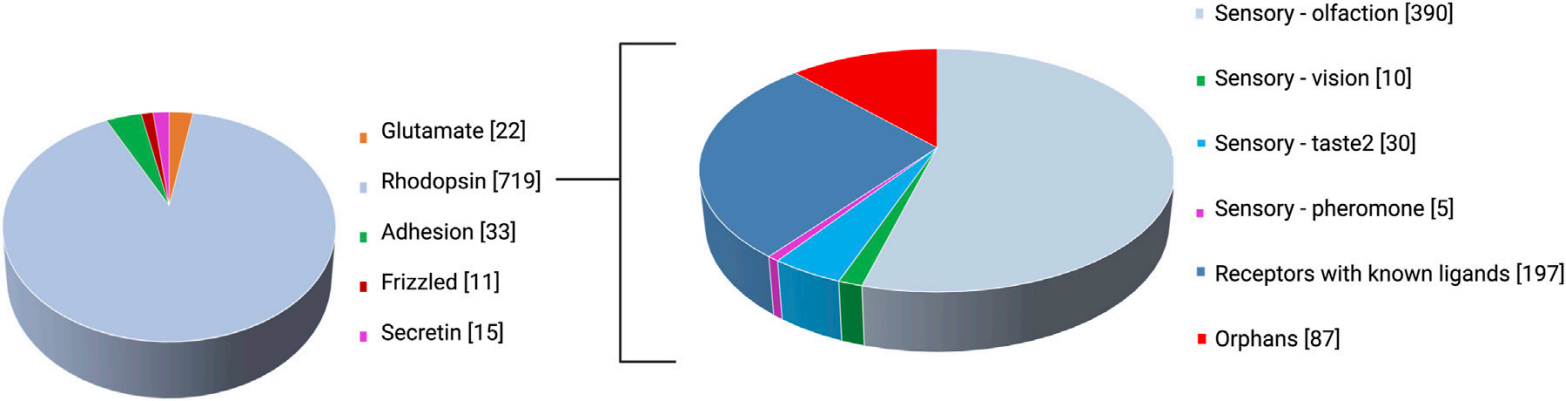
GPCR classes as per the GRAFS classification system and rhodopsin (class A) subcategorization.

G protein-coupled receptors (GPCRs) or seven transmembrane (or 7TM) receptors constitute seven transmembrane domains (TM1-7) traversing the plasma membrane; with the amino end extracellular and the carboxy terminus in the cytoplasm (Stadel et al., 1997). They make up 4% of the human genes (Kooistra et al., 2021) and nearly 13% of total membrane proteins (Muratspahić et al., 2019) and mediate the signaling of roughly two-thirds of hormones and neurotransmitters (Foster et al., 2019). Hence, the GPCR family is the largest family of membrane receptors (Matthews et al., 2017), and the most targeted given their role in modulating virtually all physiological processes (Hauser et al., 2017; Sriram and Insel, 2018). The most popular GPCR classification the GRAFS—Glutamate, Rhodopsin, Adhesion, Frizzled, Secretin—system was proposed by Fredriksson et al. (2003) on the basis of sequence similarity. The array of established GPCR ligands is as wide-ranging and rich as the physiological processes that they mediate, and these include odorants, gustatory molecules, ions, photons, protons, neurotransmitters, hormones, chemokines, lipids, pheromones, amino acids and their derivatives, peptides, nucleotides, small organic molecules (Bockaert, 1999; Civelli, 2001; Kroeze et al., 2003; Civelli et al., 2006) and microbial products such as short chain fatty acids and signal peptides (Brown et al., 2003). Despite the broad scope of GPCRs mediating physiological processes and their therapeutic relevance in cancer, metabolic disorders, autoimmune diseases, and central nervous system (CNS) disorders, some remain as orphans for which no endogenous ligand(s) have been identified (Zhao et al., 2021). Orphan GPCRs account for ∼30% of the ∼400 non-olfactory human GPCRs (Laschet et al., 2018; Alexander et al., 2019) as illustrated in Figure 1. The “non-sensory” GPCRs are targeted by over 40% of clinically administered drugs (Zhao et al., 2021). In a joint effort between the British Pharmacological Society and the International Union of Basic and Clinical Pharmacology (IUPHAR), a record of orphans and the extensive set of deorphanized GPCRs (Alexander et al., 2019) is archived in the Guide to Pharmacology database accessible at https://www.guidetopharmacology.org/GRAC/ReceptorFamiliesForward?type=GPCR. As per the IUPHAR, an orphan GPCR is deemed “deorphanized”—paired with its endogenous ligand(s)—when two or more reviewed publications from independent studies report ligand activity upon receptor binding, with a potency corresponding to biological function (Davenport et al., 2013). The list of orphan GPCRs is provided in the Supplementary Appendix SA1 (Alexander et al., 2021; Alexander et al., 2023; Behrens, 2023; Bikle et al., 2023).

The overall approach to orphan receptor characterization is dubbed the “reverse pharmacology” approach, which starts with the receptor as a biological target to uncover its cognate ligand, unique from “forward pharmacology” (Lee et al., 2003) which follows the classical drug discovery pipeline. Specifically, orphan GPCRs under study are expressed in eukaryotic cells by DNA transfection to gauge the binding efficiency of prospective ligands (Libert et al., 1991; Mills and Duggan, 1994). Considering that GPCRs are the most crucial pharmaceutical targets for therapeutic development (Kapolka et al., 2020), it is key to address the current challenges in characterizing the remaining orphan GPCRs.

The present review focuses on the techniques employed in exploring orphan GPCRs and the numerous challenges to their deorphanization.

## 2 Techniques employed in orphan GPCR deorphanization

### 2.1 Reverse pharmacology and functional screening

**FIGURE 2:**
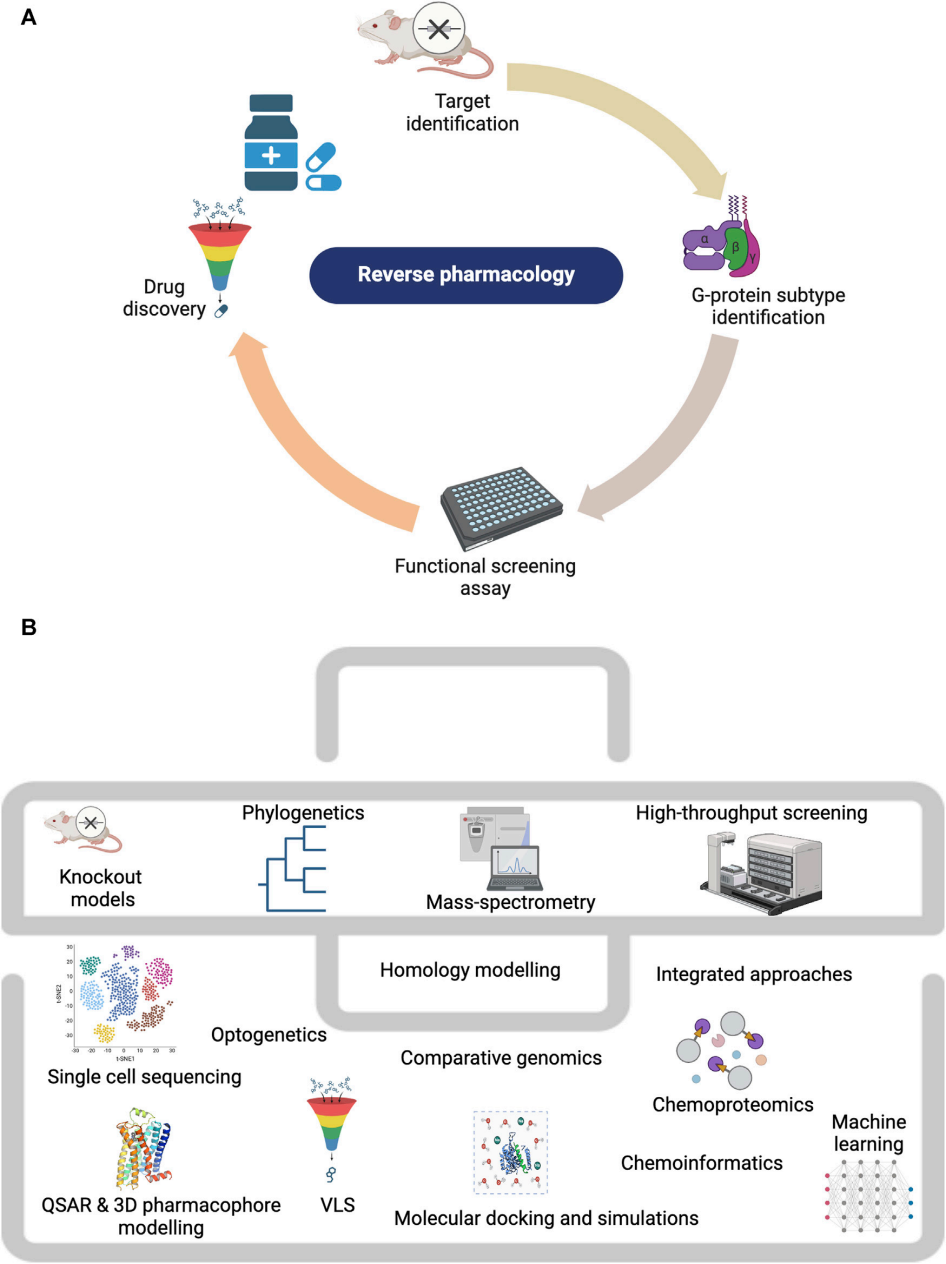
Techniques employed in studying orphan GPCRs. **(A)** The reverse pharmacology workflow. **(B)** The orphan GPCR deorphanization toolkit. Created with BioRender.com.

Reverse pharmacology was traditionally the first strategy employed for homing orphan GPCRs and resulted in the first deorphanization of two GPCRs in 1988, the serotonin 5-HT1A reported by Fargin et al. (1988) and dopamine D2 receptors by Bunzow et al. (1988). Low-stringency hybridization (Bunzow et al., 1988) which detects nucleic acids with partial homology under flexible parameters and PCR-derived techniques (Libert et al., 1991), pioneered the discovery of many GPCRs. Given its rapidity, the PCR-based approach was the preferred method for discovering novel orphan GPCRs (Civelli et al., 2006), while low-stringency screening was most popular for discovering GPCR subtypes (Chung et al., 2008). Figure 2 outlines the steps employed in the reverse pharmacology approach and the techniques used in orphan GPCR characterization. The efficient application of reverse pharmacology necessitates adequate orphan receptor expression, top-quality ligands, and reliable screening methods to measure receptor activation (Lerner, 1994; Marchese et al., 1999). Prior to orphan GPCR deorphanization, the clinical relevance of the orphan GPCR under study is first investigated (Petryszak et al., 2016). In this regard, the phenotypic characterization of knockout mouse models (Davenport et al., 2013) and receptor expression studies through *in situ* hybridization have been highly beneficial in understanding the physiological role of orphan GPCRs and in indicating their validity as prospective therapeutic targets (Stockert and Devi, 2015). Recently, advanced sequencing techniques such as single-cell RNA sequencing were used to probe orphan GPCR function in health and disease. A recent example was illustrated by Heinzelmann et al. (2022), who reported orphan GPR87 as a basal cell biomarker in idiopathic pulmonary fibrosis. Similarly, Fu et al. (2022) performed single-cell sequencing to profile orphan GPRC5B expression in mice brain. They reported GPRC5B enrichment in various brain regions and noted significant levels of the receptor in its glycosylated form. To probe the function of the same receptor in pancreatic β-cells, CRISPR-Cas9-mediated knock-down was used to downregulate its expression (Atanes et al., 2018). Following the rescue of receptor function, GPRC5B expression was linked to cell proliferation and apoptosis, presenting it as a therapeutic target for type II diabetes.

After establishing pharmacological relevance, the next step entails identifying both the binding ligand and the associated G protein. The mammalian-adapted yeast pheromone response pathway is one of the most convenient methods for assessing various G protein pathways (Brown et al., 2000). Using this assay, constitutive G protein coupling in the absence of a ligand can also be detected. It was first used to identify the G protein association of the then orphan GPR43/FFA2, which was found to constitutively couple with G_αi_ and G_αq_ (Brown et al., 2003).

An alternative to screening constitutive activity employs a transcription factor response element (RE) reporter gene assay which typically involves the cloning of a luciferase reporter construct targeted by various secondary messengers such as Ca^2+,^ cAMP, ERK1/2, and RHoA (Cheng et al., 2010). Upon activation, cAMP (G_αs_) for instance induces luciferase gene transcription through the cAMP response element (CRE). The signaling mechanism of the G_αs_-coupled adhesion receptor GPR133 was revealed through this approach (Bohnekamp and Schöneberg, 2011). More recently, G protein coupling of unliganded orphan GPCRs—GPR22, GPR137b, GPR88, GPR156, GPR158, GPR179, GPRC5D and GPRC6A—with pathophysiological association were accurately detected using luciferase reporter assays (Watkins and Orlandi, 2021). Calderon-Zamora and others developed an online platform ‘PRED PAR2.0’ for the *in silico* prediction of receptor-associated G protein subtype for former orphan receptors GPR99 and GPR107 (Calderón-Zamora et al., 2021). Following G protein coupling, conventional functional screening assays including GTP binding, calcium release, radio-ligand binding, and cAMP level modulation (Pausch, 1997; Howard et al., 2001; Bates et al., 2006; Bikkavilli et al., 2006; Overton et al., 2006; Suga and Haga, 2007; Crouch and Osmond, 2008; Tang et al., 2012) should be performed to test ligand-induced receptor activation. The chemical Similarity Ensemble Approach (SEA) approach, a target prediction model is a promising method for evaluating the probability of ligand binding to a given GPCR (Keiser et al., 2009). This approach could prove useful as an initial tool to probe ligand-receptor complementarity.

By the early 90s, numerous GPCRs were pharmacologically characterized through the reverse pharmacology approach, and high-throughput assays monitoring second messenger responses were developed by the mid-1990s; enabling reverse pharmacological methods grounded on receptor activation rather than mere receptor binding. In terms of orphan GPCR ligands, initial efforts focused on identifying novel neuropeptides since orphan GPCR expression was located primarily in the brain (Chung et al., 2008). Early attempts at orphan GPCR deorphanization even included phylogenetic analysis of known GPCRs for ligand prediction, on the premise that homologous receptors have earlier proven to accommodate agonists with analogous structural traits (Joost and Methner, 2002). Such an approach revealed an evolutionary relationship of orphan receptors to deorphanized ones with appreciable significance values.

### 2.2 Structural developments toward GPCR characterization

GPCR deorphanization hit its pinnacle in the latter part of the 1990s into the early 2000s, owing to the convergence of industry funding, the advent of high-throughput reverse pharmacology strategies, and human genome sequencing with ∼10 annual deorphanization reports. Some receptors such as bombesin receptor-3 (BRS-3) proceed through clinical trials even prior to deorphanization (Reitman et al., 2012). The year 2000 marked a great milestone with the publication of bovine rhodopsin, the first GPCR structure (Palczewski et al., 2000). The following GPCR structure was of human β2-adrenergic receptor 7 years later due to the challenges in protein crystallization (Cherezov et al., 2007). Since then, advances in crystallization methods propelled a sharp rise in the number of available GPCR structures (Stockert and Devi, 2015). This pushed the transition from conventional high-throughput screening (HTS) to virtual ligand screening (VLS) techniques for the discovery of novel GPCR ligands (Ngo et al., 2016). AlphaFold, a recent deep learning-based protein structure predictor shows promise in predicting the tertiary configurations of all proteins, including transmembrane proteins (Senior et al., 2020). Given the scarcity of GPCR crystal structures, AlphaFold-predicted structures can be used in structure-based approaches, ideally with improvements in tertiary structure-independent analysis or sequence similarity evolutionary relatedness (Satake et al., 2023). AlphaFold2 (Jumper et al., 2021) and RoseTTAFold (Humphreys et al., 2021) can predict the 3D structures of input sequences with incredibly high accuracy to improve the reliability of *in silico* orphan GPCR structure prediction.

Structural developments offer insights into 3D receptor structure, ligand and allosteric binding pockets; and conformational dynamics which is key for unravelling the mode of receptor activation as illustrated by Weis and Kobilka (2008) who elucidated the activation mechanism of rhodopsin through successive crystallization of the ligand-bound receptor in various active and inactive conformations. Most recently, Wong et al. (2023) resolved a high-resolution structure of G_αs_-coupled orphan GPR21 through cryo-electron microscopy (cryo-EM) and single-particle analysis. Structure-directed mutagenesis along with biochemical analysis revealed constitutive signaling of GPR21 via G_αs_-coupling. Similarly, the high-resolution structures of human orphan GPR52 in different states—ligand-free, G_s_-coupled, allosteric-bound ligand—unraveled the mechanism of ligand recognition and self-activation (Lin et al., 2020).

Additionally, crystal structures serve as a template for the homology modelling of closely related receptors. For instance, the crystal structure of the adenosine A (2A) receptor was used to model GPR55 for ligand docking (Elbegdorj et al., 2013). Homology models have also been used to examine the effect of structural mutations as demonstrated by Shimomura et al. (2008) with former orphan receptor P2RY5. However, homology modelling is limited in handling loop regions which are poorly conserved between GPCRs and are crucial in ligand binding and receptor activation (Goldfeld et al., 2011). With advancements in machine learning approaches, there has been great progress in 3D structure prediction. Recently, Wang et al. (2023) comparatively assessed the predictive performance of 13 loop modelling approaches on protein loops spanning 4 to 69 residues—popular deep learning algorithms AlphaFold2 and RoseTTAFold, eight *ab initio* methods (Modeller, DISGRO, GalaxyFill, KIC, CCD, NGK, RML and Remodel), and three knowledge-based methods (Prime, FREAD, and MOE search). Using 10,423 loop structures from the Protein Data Bank (PDB) and 549 datasets from the Critical Assessment of Structure Prediction (CASP) library, they reported AlphaFold2 and RoseTTAFold to show great potential in predicting loops longer than 16 and 30 residues in the CASP and PDB datasets, respectively. Structural studies also reveal post-translational modification (PTM) moieties in high-resolution structures. For instance, a rhodopsin-arrestin complex crystallized through X-ray free electron laser (XFEL) revealed two phosphorylated sites—Thr336 and Ser338 (Zhou et al., 2017). Additionally, palmitoylation at cysteine residues was reported in structures of both rhodopsin and β2-adrenoceptor (Salom et al., 2006; Cherezov et al., 2007).

### 2.3 Structural and ligand-based approaches

Through the years, orphan receptor ligand discovery was facilitated by an array of techniques including 1) demonstrating a link between ligand and receptor expression profile, 2) evaluating tissue extracts in receptor-guided functional screening, 3) testing agonists for characterized GPCRs on orphan GPCRs with significant sequence homology, and 4) arbitrarily testing of orphan GPCRs against a set of well-established ligands (Metpally and Sowdhamini, 2005). With the availability of GPCR crystal structures, there have been several exemplary cases of GPCR ligand discovery through molecular docking, the most fruitful studies targeted receptors with orthosteric binding cavities (Ballante et al., 2021) such as adrenergic, adenosine, dopamine, serotonin, muscarinic, metabotropic glutamate (mGlu1 and mGlu5), histamine, and melatonin receptors.

Through virtual screening of large chemical libraries, structure-based molecular docking cuts time and resources by shortlisting candidate molecules for experimental testing. Additionally, the prediction of potential ligand binding sites via molecular docking guides experimental efforts in the validation of catalytic sites and in elucidating the molecular basis of receptor activation. Such an approach resulted in the characterization of the β2-adrenergic receptor, the target of the first effective docking screening against a GPCR crystal structure (Kolb et al., 2009). Following the docking of chemical libraries with “lead-like” compounds against the crystal structure (PDB ID 2RH1), the top 25 molecules were shortlisted for further testing in a radioligand displacement assay to measure ligand activity. Similarly, structure-guided VLS saw great success in the discovery of novel ligands (Qin et al., 2015; Ngo et al., 2016). For instance, the internal coordinate mechanics (ICM) approach integrates a VLS algorithm that flexibly docks millions of potential ligands despite improper sidechain positions, as this can be corrected through the ICM global optimization with great precision regardless of a bound or unbound ligand (Cavasotto et al., 2003). This was the first study showcasing the feasibility of VLS for orphan GPCR deorphanization.

In the absence of experimental structures as recently reported for up to 94% of GPCRs (Schneider et al., 2018), homology models which were originally and most commonly applied to identifying exogenous ligands for therapeutic development can be exploited; as such models have proven to be up to par with crystal structures in terms of hit rates in virtual screens (Carlsson et al., 2011). It is noteworthy that the optimal homology model template for an orphan GPCR does not need to necessarily belong to the same receptor subclass (Kroeze et al., 2015), as varying subclasses may exhibit comparable modes of binding (Kufareva et al., 2011; Rataj et al., 2014). The identification of modulators for the previously orphan GPCRs MAS- related GPR family member X2 (MRGPRX2), GPR65, and GPR68 are exemplary cases of docking screens that discovered ligands using homology models built around remote templates (Huang et al., 2015; Lansu et al., 2017).

3D pharmacophore modelling is another element of the GPCR deorphanization toolkit. Structure-guided pharmacophores were first designed for the β2-adrenoceptor and GPR40/FFA1 for novel ligand discovery (Tikhonova et al., 2007; Barillari et al., 2008). However, translating structure-guided pharmacophore modelling to orphan GPCRs is constrained by unrefined binding cavities (Ngo et al., 2016) just as ligand-guided pharmacophores is limited by ligand scarcity. For instance, an attempt made by Isberg et al. (2014) unveiled aromatic L-amino acids as probable GPR139, native ligands, however, GPR139 remains an orphan receptor (Pallareti et al., 2023). Nonetheless, logically, ligand-based pharmacophores should be developed in tandem with structure-based ones (Ngo et al., 2016). Other ligand-based strategies such as quantitative structure-activity relationship (QSAR) models built around a knowledge base of ligands can offer insights into ligand-receptor interaction (Acharya et al., 2011). Of recent, Noonan and others detailed the use of 3D pharmacophore modeling as an initial step for the identification of allosteric and biased ligands, GPCR deorphanization, elucidation of GPCR pharmacology and visualization of ligand-receptor interaction patterns among other applications (Noonan et al., 2022).

Moving forward, integrating 3D pharmacophore models with molecular dynamics simulations and advanced techniques like machine learning would offer great insight into orphan GPCR pharmacology.

### 2.4 Integrative approaches

Expectedly, integrative approaches to orphan GPCR deorphanization saw much greater success relative to single functional assay-based approaches. A successful case study was demonstrated by Huang et al. (2015) in deorphanizing the pharmacologically dark receptor orphan GPR68. Using yeast-based screening and computational modeling, the researchers identified lorazepam as a positive allosteric modulator (PAM) of GPR68. Further optimization through docking of over 3 million available lead-like molecules against the putative lorazepam binding site led to the discovery of ogerin, a more potent PAM. To showcase the applicability of their integrated approach, they successfully reported ligands for GPR65, a related proton-sensing GPCR with 37% sequence homology. To explore the nature of human peptidergic signaling systems (peptide-receptor signaling), Foster et al. (2019) integrated bioinformatics for the structural assessment of human class A GPCRs and comparative genomics spanning 313 species. Through a machine learning strategy, namely, logistic repression, they predicted a candidate set of 21 peptide-binding human class A orphan GPCRs based on universal characteristics of peptidergic signaling systems. These features were subsequently used to mine putative peptide ligands from the vast secreted human proteome through a proteome-wide machine learning approach which identified a library of 218 peptides. The group reported peptide ligands for five orphan receptors—BB3, GPR1, GPR15, GPR55, and GPR68 via multiple pharmacological screening assays—mass redistribution, receptor internalization and β-arrestin recruitment, inositol phosphate accumulation, cAMP inhibition and calcium assay—to cover various signaling pathways. This study represents a breakthrough in orphan-GPCR deorphanization through the application of machine learning-assisted strategies. They also compiled a ligand set comprising 1,227 ‘‘cleavage variants’’ as a repository for prospective ligands to facilitate future efforts at homing orphan GPCRs. Similar to secretome screening performed by Foster’s group, metabolome screening constitutes another approach to GPCR deorphanization since microbiota-produced metabolites can act as ligands of well-characterized GPCRs and orphan GPCRs alike (Chen et al., 2019). Other integrated approaches such as chemoinformatics which draw upon ligand resources such as QSAR, pharmacophore models, docking, and molecular dynamics (MD) simulations to explore the function of chemical networks (Kumar et al., 2021) also hold promise for orphan GPCR deorphanization. This was exemplified by Jacob and others through integrated *in silico* chemogenomics with machine learning. Their integrated technique surpassed earlier ligand-based strategies in terms of interaction prediction accuracy for receptors with known ligands and orphans alike. Given no receptor 3D structural data, their method predicted orphan GPCR ligands with an accuracy of about 78.1% (Jacob et al., 2008). More recently, Velloso et al. took a chemoinformatics approach in using graph-based signatures to launch the pdCSM-GPCR webserver for the prediction of GPCR ligand bioactivity for the largest set of human GPCR types, including two orphans. Their model far exceeds earlier approaches and represents the most extensive computational tool for predicting GPCR bioactivity presently. The pdCSM-GPCR workflow includes dataset acquisition, feature engineering, and machine learning. Briefly, experimental data on the ligands of 36 GPCRs was retrieved from PubChem and features of these ligands were assigned to model different facets of ligand-receptor binding. They developed a machine learning algorithm that uses computed features and bioactivity data as evidence to train, test, and validate predictive models through supervised learning. Given no structural input, they report that potent GPCR ligands typically possess aromatic bicyclic rings, aromatic rings, and nitrogen-containing fragments (Velloso et al., 2021). Most recently, Zhao et al. (2023) adopted a chemoproteomics approach to characterize the human protein targets of microbial metabolites. Through mass spectrometry-based proteomics, orphan GPRC5A was identified as a hit. Microbial monoamine indole metabolites were found to activate the receptor, they associated this agonism with pathways related to immune response and cancer signaling.

Uniquely, other integrated approaches involve bypassing endogenous ligand activity through the optical functionalization of receptor signaling. An example of this was demonstrated by Zheng et al. (2018) who used the light-gated cation channel Channelrhodopsin 2 (ChR2) as an optogenetic tool to optically activate orphan GPR37. They engineered the ChR2-GPR37 chimera by replacing the intracellular loop sequences of the cation channel with corresponding regions of the receptor. Upon photoactivation, the ChR2-GPR37 construct triggered characteristic GPR37 signaling, denoted by lower cAMP levels, increased ERK phosphorylation and higher motor activity *in vivo*. Interestingly, this technique also unveiled novel facets of GPR37 pharmacology such as IP3 signaling and an anxiety-like response in animal models. Additionally, elevated levels of IP1 suggest GPR37-mediated G_q_ signaling. As an emerging target for Parkinson’s disease (PD), the ChR2-GPR37 chimera can be used to further explore this possibility in PD cell types. Through a similar optical approach, the previously classified human pseudogene GPR33 was functionally resurrected (Morri et al., 2018), with observed signaling pathways including Ca^2+^, cAMP, MAPK/ERK, and Rho-dependent pathways, reinforcing its supposed role in pathogen entry.

### 2.5 Machine learning and prediction-based approaches

Presently, reverse pharmacology remains the leading strategy for GPCR deorphanization and is now integrated with “high-throughput” signaling detection technologies, leveraging the availability of genomic, transcriptomic, metabolomic and/or peptidomics data. Yet, such techniques are expensive, laborious, and inconsistent, hence demanding multiple transfections and signal monitoring. Justifiably, the past decade saw a decline in the frequency of orphan GPCR deorphanization, in addition to retracted pairings due to failed reproducibility, indicating the limits of the current high-throughput approaches. It is, therefore, necessary to place more effort into systematic predictions of ligands and GPCRs (Satake et al., 2023), as offered by machine learning approaches which perform a systematic prediction of ligand-GPCR interaction. Unlike bioinformatics and computational analyses which are mostly based on sequence similarity and/or tertiary structures of peptides and GPCRs, machine learning techniques work with comparatively less peptide and GPCR sequence data, as well as established empirical data associated with peptide-GPCR interactions. Additionally, the high accuracy of machine learning techniques facilitates time-efficient experimental validation of new peptide-GPCR pairings. Uniquely, the “prediction-experimental validation-data feedback” loop aspect of machine learning substantially boosts the systematic and effective prediction of novel peptide-GPCR interactions; as feedback on experimental outcomes for predicted data enhances prediction accuracy. Moreover, machine learning techniques are anticipated to reveal hidden molecular patterns of peptide-GPCR pairs and evolutionary mechanisms beyond the scope of strategies relying on sequence homology and molecular phylogeny (Satake et al., 2023). Machine learning was first employed to predict ligand-GPCR interactions by Weill and Rognan (2009). GPCR descriptors were computed based on donor-acceptor pairs, electric charge, hydrophobicity, molecular weight, and aromaticity of the residues within the receptor catalytic site, while ligand descriptors were extracted from MACCS keys and SHED descriptors (molecular representations encoding ligand structural and chemical data). The machine learning model used as input 32,118 established ligand-GPCR interactions and 202,019 non-complementary pairs. Model validation reported about 85% predictive power for established ligand-GPCR interactions, highlighting the promise of machine learning for the prediction of ligand-GPCR pairs. The earliest machine learning-based prediction of novel ligand-GPCR pairing for known small molecules and GPCRs with 91.9% ± 0.3% accuracy was reported by Yabuuchi et al. (2011) who used chemical genomics-based virtual screening (CGBVS), a computational screening approach to identify novel scaffold-hopping compounds. The binding prediction between the β2-adrenoreceptor and 11,500 commercialized small molecules followed by experimental assays identified nine new ligands. Additionally, three novel ligands were identified for neuropeptide Y receptor 1. Interestingly, some of these compounds exhibited chemical structures distinct from those of established agonists and antagonists of both receptors. Numerous studies followed, showcasing the application of machine learning for the efficient and systematic prediction of novel GPCR-ligand pairs with high accuracy. For instance, drawing on ligand structural data and GPCR amino acid motif sequences as opposed to receptor 3D structure, Seo et al. (2018) proposed a GPCR-ligand binding prediction model which predicted ligands for 100 unpaired GPCRs selected at random from the GPCR-Ligand Association (GLASS database) with predictive power of 0.94. Some of the matched ligand-GPCR pairs were corroborated by multiple studies. More recently, Oh et al. (2022) formulated a ligand-based machine learning model for the prediction of GPCR-ligand interaction primarily for application in drug discovery. They took a two-model prediction approach where the first model identifies GPCR-binding ligands and the second categorizes the ligands as agonists or antagonists. Given 990 predictor features from 5,270 molecular descriptors (ligand chemical and physical properties) calculated from 4,590 ligands archived in two drug databases, the model predicted agonists, antagonists and non-ligands with 0.733 accuracy. Following model validation with FDA-endorsed GPCR drugs, 70% of these drugs were successfully categorized as agonists or antagonists.

Similar to homology modeling, machine learning models typically fail with unannotated proteins exhibiting poor sequence homology with known proteins. To overcome this limitation, Cai et al. (2021) developed distilled sequence alignment embedding (DISAE) to represent protein sequences for the prediction of chemical binding to evolutionary divergent unannotated proteins through deep learning. DISAE can identify functional links between proteins given neither structural nor functional input. As such, it was able to predict pairings of orphan receptors with approved drugs.

### 2.6 Developments in screening-based strategies

Back in 2015, Kroeze et al. devised Parallel Receptorome Expression and Screening via Transcriptional Output—TANGO (PRESTO-TANGO), an open-source tool based on β-arrestin recruitment to explore the druggable human GPCRome (Kroeze et al., 2015). Most recently, Zeghal’s group developed TANGO-trio, an evolved comprehensive high-throughput cell-based platform to profile basal and ligand-dependent GPCR activity in parallel. Through this platform, they reported induced basal activation curves at about 200 rhodopsin GPCRs, including over 50 orphans. Most importantly, this approach sets apart constitutive and ligand-induced activation mechanisms, as well as state-independent activation (Zeghal et al., 2023). Similarly, Morfa et al. (2018) developed a unique high-throughput cell-based screening method for orphan GPCR deorphanization drawing on the concept of pharmacochaperones, in which cell-permeable small compounds enable mutant receptor trafficking to the plasma membrane. In combination with a β-galactosidase reporter system, molecules acting as pharmacochaperones to facilitate the forward trafficking of the mutant GPCR target can be identified. As a proof-of-concept, this approach was applied to the β2-adrenergic receptor to probe its already identified ligands; and was able to successfully set apart agonists and antagonists. However, not all receptors can be characterized through this technique, particularly non-class 2 orphans where the altered receptor sequence may not be well-conserved. Additionally, this method will prove ineffective if induced mutation results in a substantial loss of receptor tertiary structure or jeopardizes sustained trafficking to the ER.

Other recent efforts include the development of yeast-based Dynamic Cyan Induction by Functional Integrated Receptors (DCyFIR) technology for high-throughput CRISPR engineering and GPCR ligand characterization to unlock the potential of poorly studied GPCRs. This system profiled 320 human metabolites and revealed numerous GPCR-metabolite associations, many of which were related to unexplored ‘pharmacologically dark’ receptors—GPR4, GPR65, GPR68, and HCAR3. Due to the simultaneous screening of ligands against several receptors, DCyFIR profiling allows for the physical testing of a massive number of substances, bridging the gap between wet-laboratory and *in silico* studies (Kapolka et al., 2020).

Analogous to orphan receptors, the native receptors of secreted orphan ligands remain unidentified, though numerous drugs target such ligands and their receptors. Cell-based screening is also an attractive method for the characterization of extracellular orphan ligand-receptor interactions as multimerized ligands can compensate for cells expressing poor-affinity cell surface receptors. Following biochemical validation, this method revealed several novel ligand-receptor pairs including receptor tyrosine phosphatase ligands and interactions having implications for immune system function (Siepe et al., 2022). This technique may be transferable to orphan GPCR deorphanization considering that orphan lipids N-arachidonoyl glycine and farnesyl pyrophosphate were reported to act at GPR18 and orphan GPR92, respectively (Bradshaw et al., 2009).

## 3 Challenges in orphan GPCR deorphanization

### 3.1 Ligand availabilit*y*

Although numerous bioactive peptides have been discovered through mass spectrometry (Hauser et al., 2020), ligand availability remains the major barrier to orphan GPCR deorphanization. Additionally, the presence of an orphan GPCR away from its ligand’s synthesis site presents another hurdle. Moreover, the possibility of multi-ligand binding may also hinder deorphanization efforts. Furthermore, orphan GPCR functionality could also present a hindrance as some receptors are only functional within a heterodimer (Kaupmann et al., 1997), while others require accessory proteins and metal ions as cofactors for their activation (McLatchie et al., 1998). While some GPCRs with just 25% similarity can engage a common ligand (such as the histamine receptors), others with higher homology (such as the melanocortin, lysophosphatidic acid, and sphingosine 1-phosphate [S1P] receptors) may necessarily not (Gaulton, 2003). This suggests an unpredictable nature of ligand binding. Ideally, the discovery of a receptor’s signaling network should be the starting point in the search for its ligand (Laschet et al., 2018). Subsequently, a suitable assay (Chung et al., 2008) should be carried out to ensure that receptor-ligand interaction elicits a response. Additionally, the nature of endogenous ligand pharmacology can present a further challenge (Laschet et al., 2018). Though ligands are often regarded as agonists, they could also be inverse agonists (Nijenhuis et al., 2001) or even antagonists (Ballante et al., 2021). Interestingly, the activation of epithelium 5-HT4 receptors by tryptamine, a microbial metabolite was reported to mediate secondary messenger pathways (Bhattarai et al., 2018). Therefore, a ligand source that merits future efforts to pair the remaining orphan GPCRs include microbiota-secreted metabolites, signal peptides and short-chain fatty acids (Dierking and Pita, 2020), fatty acid amides (FAAs) (Chang et al., 2021), end products of polysaccharide fermentation such as butyrate and pentanoate (Samuel et al., 2008) among others. Examples of other GPCRs—some of which are orphans–that respond to bacterial metabolites include GPR41 and GPR43, GPR109a, the four identified histamine receptors (H1R-H4R), GPR139 and the CaS receptor, GPR51, GPR17, GPR105, P2Y receptors (Forde et al., 2022), GPR119 and orphan GPR132 (Chang et al., 2021). Consequently, the screening of microbial genomes would be valuable for uncovering new ligands. To facilitate the prediction of novel GPCR ligands, Genepep established a specialized bioinformatics platform to scan transcriptome databases. Such effort led to the characterization of the QRFP/P52 peptide (Colette et al., 2007).

**FIGURE 3:**
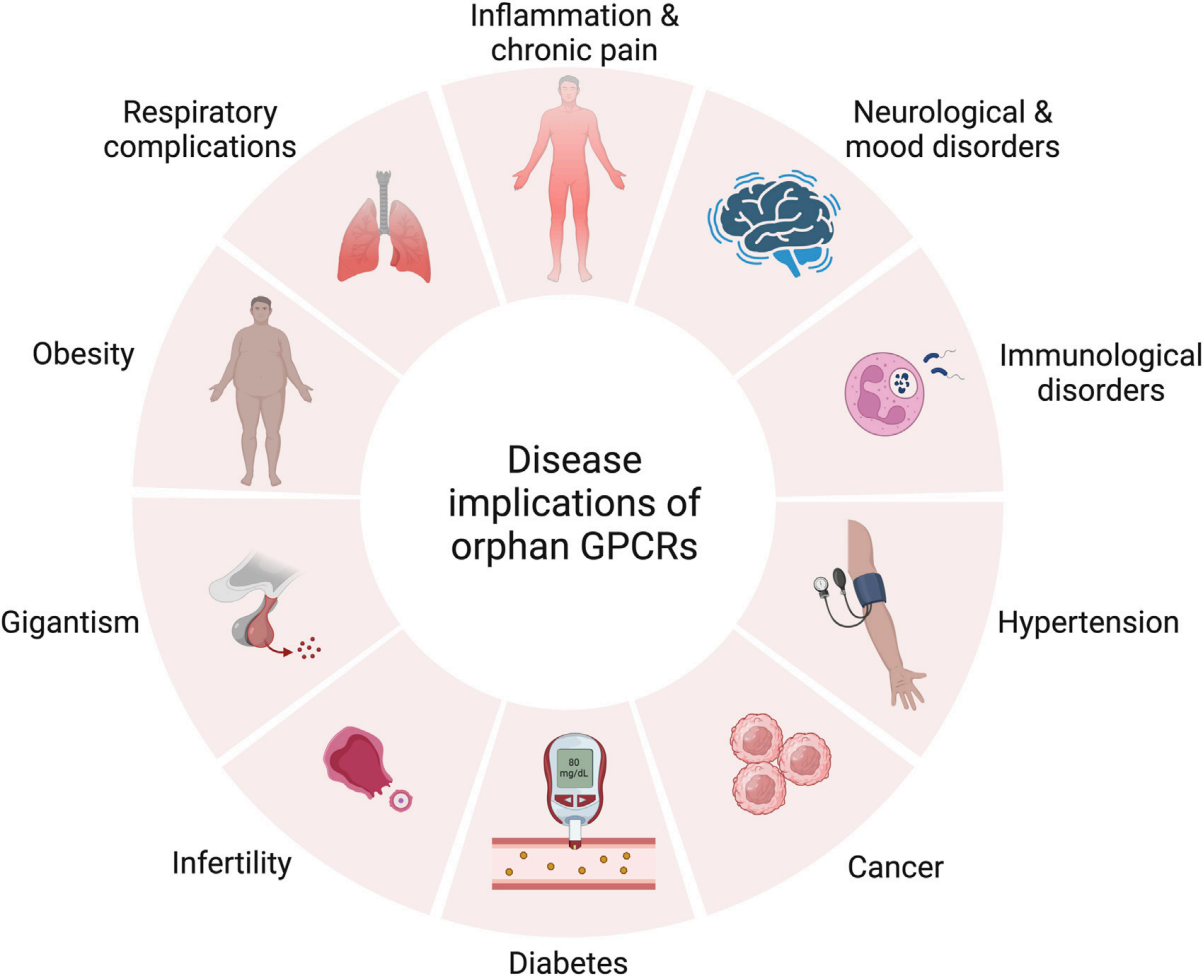
Therapeutic relevance of orphan GPCRs in various diseases. Created with BioRender.com.

As illustrated in Figure 3, orphan GPCRs have been linked to various diseases in numerous studies (Drevets et al., 1997; Fujiwara et al., 2007; Trivellin et al., 2014; Luo et al., 2015; Cui et al., 2016; Honn et al., 2016; Ricaño-Ponce et al., 2016; Stein et al., 2016; Calderón-Zamora et al., 2017; Chen et al., 2017; Li et al., 2017; Wang et al., 2017; Guns et al., 2018; Nourbakhsh et al., 2018; Yang et al., 2021).

In view of the therapeutic relevance of orphan GPCRs in various diseases, drug discovery efforts following deorphanization can benefit from the striking structural and functional range of natural peptides sourced from plants, bacteria, and fungi; along with venom-derived peptides isolated from snakes, cone-snails, scorpions, and spiders. This is owing to their drug lead-like features being distinct from those of synthesized small molecules, making them a valuable prototype for novel GPCR ligand design (Muratspahić et al., 2019). Synthetic surrogate ligand discovery for orphan GPCRs offers a compelling alternative to traditional deorphanization methods (Jacob et al., 2008). However, such ligands may elicit biased signaling, inducing receptor conformations that support an entirely distinct downstream signaling cascade. Thus, they should be complementary, and not supplementary to native ligands (Ahmad et al., 2015).

### 3.2 Limitations of current strategies

Besides ligand availability, the declined deorphanization frequency (Levoye and Jockers, 2008) could be related to the limitations of existing strategies in covering the unconventional GPCR signaling pathways. The earliest attempts at homing orphan GPCRs explored the use of non-mammalian systems. The natural mechanism in yeast where pheromone-responsive GPCRs initiate mating was adapted to study mammalian orphan GPCRs since GPCR signaling in human cells resembles that of *Saccharomyces cerevisiae*. The first successful yeast screen identified isoproterenol as a non-selective ligand of the β2-adrenoceptor (King et al., 1990). Another successful deorphanization using this strategy was that of former orphans GPR41 and GPR43 activated by propionate and short-chain carboxylic acids (Brown et al., 2003). However, only about 60% of mammalian GPCRs exhibit functionality in yeast, possibly due to low receptor expression or challenges in coupling to chimeric G proteins (Dowell and Brown, 2002). Additionally, mammalian-based *in vitro* screens may fall short in cases where cofactors or post-translational modifications are critical for receptor activation (Qanbar and Bouvier, 2003). Moreover, the reverse pharmacological method targets alterations in second messenger levels assuming the prior understanding of receptor pharmacology (Chung et al., 2008); which for certain GPCRs can be unpredictable as peculiar GPCR pathways involving ligand-independent signaling pose another challenge. Besides G proteins and β-arrestins, various other GPCR-interacting proteins can influence receptor function. Therefore, it is vital to identify the entire set of GPCR-interacting proteins (Ritter and Hall, 2009). Functional screens typically monitor intracellular second messengers of G protein-dependent pathways while some receptors are G protein-independent (Lefkowitz and Shenoy, 2005). An example is GPR77 which in binding assays exhibits interaction with C5a anaphylotoxin while exhibiting no activity in G protein-dependent assays (Cain and Monk, 2002; Kalant et al., 2003). Additionally, β-arrestin screens for instance only identify agonists (Milligan, 2003a) and hence would fail in capturing endogenous inverse agonists, biased and allosteric agonists. Biased agonism illustrates one facet of GPCR pharmacological complexity, where a ligand selectively activates a subset of downstream pathways. Generally, this phenomenon alludes to preference toward either the G protein or the β-arrestin pathway; but also includes G protein subtype selectivity or GPCR kinase association (Rajagopal et al., 2010). Biased signaling has also been reported between β-arrestin 1 and 2 (Hodavance et al., 2016), as well as different states of the same GPCR bound to different agonists. Alternatively, allosteric modulators can also elicit biased agonism (Lane et al., 2017) as exemplified by the allosteric modulator Org27569 with the CB1 cannabinoid receptor where Org27569 attenuated cAMP inhibition mediated by cannabinoid ligands while exerting minimal effect on ERK1/2 phosphorylation induced by some of the same ligands (Khajehali et al., 2015). Additionally, GPCRs such as adrenergic receptors engage multiple endogenous agonists, namely, epinephrine and nor-epinephrine. Similarly, several chemokines act at a group of chemokine receptors and propagate varied signaling routes pathways, highlighting the signaling bias of native ligands (Corbisier et al., 2015). However, existing assays are restricted in detecting a single signaling pathway and fail to capture such a complex phenomenon. Through a multi-assay approach, both G protein and β-arrestin–biased agonists of the sphingosine-1-phosphate (S1P1) receptor were successfully differentiated by Zhu et al. (2014). They used a high-content assay to monitor the redistribution of GFP-tagged β-arrestin and an aequorin assay to detect alterations in intracellular Ca^2+^ levels. To follow up on compounds exhibiting bias across the two platforms, the group took to more conventional GPCR screening assays including a competitive radioligand binding assay, cAMP accumulation assay, GTPγS binding assay, and an alternative β-arrestin redistribution assay (β-arrestin Tango). Interestingly, they identified a compound that elicits an unusual pattern of β-arrestin translocation and GPCR recycling dynamics.

Furthermore, considering that several GPCRs exhibit multiplicity in G protein coupling (Hermans, 2003), conventional single-platform assays fail to offer comprehensive coverage of receptor pharmacology. This could justify the low hit rate in a β-arrestin recruitment screening assay conducted by Southern’s group where the screening of 5,300 putative endogenous agonists against 82 orphan receptors reported proposed ligand fatty acids for only one orphan—GPR84 (Southern et al., 2013). Though calcium mobilization assays are popular for orphan GPCR deorphanization, this platform fails to capture elevated basal Ca^2+^ in the context of constitutively active G_q_-coupled receptors (Robas and Fidock, 2005). Likewise, the guanine nucleotide binding assays which measure [^35^S]GTPγS are widely used since guanine nucleotide exchange is closely followed by receptor activation. This assay is however typically confined to G_i/o_-coupled receptors, with G_i/o_ being the predominant G protein in most cells (Milligan, 2003b). Additionally, it generates limited throughput due to a filtration step to isolate [^35^S]GTPγS in its free and bound states (DeLapp, 2004). cAMP assays are suitable for detecting cAMP or adenylyl cyclase activity of G_s_ and G_i/o_-coupled receptors (Gabriel et al., 2003). This platform was used to characterize orphan GPR87 as a lysophosphatidic acid receptor (Tabata et al., 2007), but similar to other assays, it is not universal and is more effective with stable G_i/o_ coupled receptors, as opposed to transiently expressed ones (Hosoi et al., 2002). Another system exploring GPCR characterization takes advantage of GPCR internalization (Koenig and Edwardson, 1997). In such systems, GPCRs are fluorescently tagged to monitor receptor translocation. This platform is low-throughput and is challenging to automate, but most importantly, gives misinterpreted results. For instance, the Mas oncogene was initially characterized as an angiotensin receptor (Jackson et al., 1988), but following studies associate the Mas receptor with modulatory function (Ambroz et al., 1991; Halbach et al., 2000).

A receptor cannot be experimentally confirmed to have no native ligand as the lack of its identification is not proof of its absence. This justifies the use of tissue extracts—the source of native ligands—in assaying receptor activity. The possible limitation of this method lies in the challenging isolation of endogenous ligands that are tightly controlled, highly unstable, transiently produced or minimally expressed ligands. The aforementioned also explains the challenge of isolating and characterizing unknown transmitters (Laschet et al., 2018).

In VLS studies, non-refined binding pocket residues of orphan GPCR homology models limit hit rates (Ngo et al., 2016). For instance, compared to the unrefined crystal structure (20%), a refined dopamine D3 receptor structure was more effective (56%) at identifying novel D3 ligands (Carlsson et al., 2011; Lane et al., 2013). In this regard, molecular dynamics simulation approaches can be used to improve the prediction of ligand binding poses for low-resolution membrane protein homology models (Schneider et al., 2018). Similarly, certain measures can be employed to improve hit rates in molecular docking studies (Wei et al., 2020; Ballante et al., 2021), such as a custom screening library to bias the identification of selective ligands or the use machine learning models to predict compounds with properties similar to those of selective ligands (Rataj et al., 2014). Additionally, ligand-based strategies can be implemented to exclude compounds related to the ligands of the anti-target from the library (Weiss et al., 2013; Ballante et al., 2021). Furthermore, known selective ligands can be docked to elucidate the basis of selectivity (Katritch et al., 2011; Ranganathan et al., 2015).

### 3.3 Irreproducible ligand-receptor pairings

The failed reproducibility of ligand-receptor pairings also explains the slow deorphanization rate of orphan GPCRs. For instance, orphans GPR32 and GPR37L were previously paired with resolvin D1 and head activator peptide, respectively. However, both pairings proved irreproducible (Laschet et al., 2018). Retraction reports of irreproducible ligand-receptor pairs share several common observations. Firstly, receptor activation is mostly assessed with a single technique, although using at least one but preferably two orthogonal assays is the benchmark when characterizing a novel ligand. Multiple GPCR signaling pathways should be timely examined upon receptor activation. Accordingly, reporter genes are discouraged since they report events far from receptor activation. Secondly, the reported proposed ligands exhibit no effect on non-transfected cells. When omitting background is technically demanding, such as in ligand-receptor ‘promiscuity’, antagonists and surrogate agonists can serve as controls. The use of cellular backgrounds that are further distant from humans and mammals, such as yeast (Liu et al., 2016), is another potential strategy for future studies aiming to revise irreproducible ligand-receptor pairings.

### 3.4 Unconventional GPCR signaling

**FIGURE 4:**
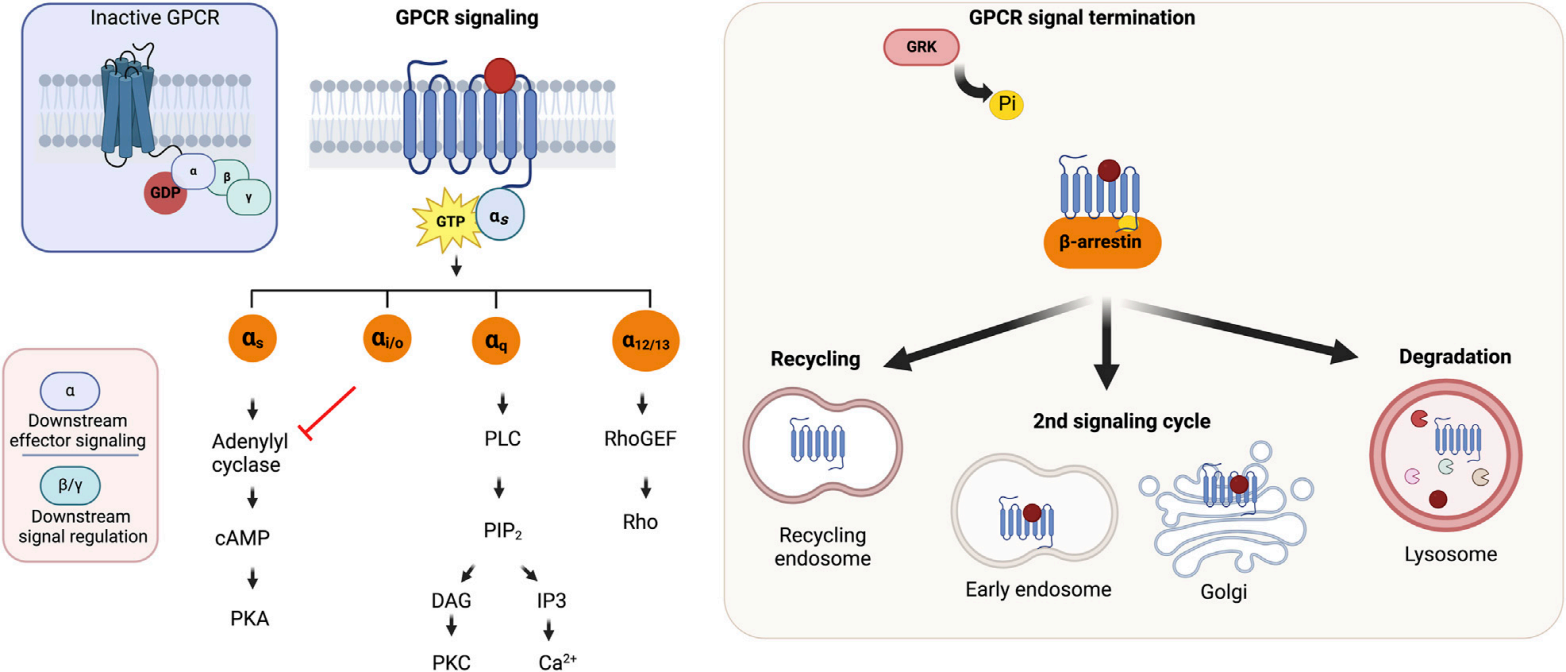
GPCR architecture and signaling. **(A)** A scheme of GPCR structure **(B)** GPCR signaling mechanism and signal termination. Adapted from Calebiro and Godbole, 2018. Created with BioRender.com.

**FIGURE 5:**
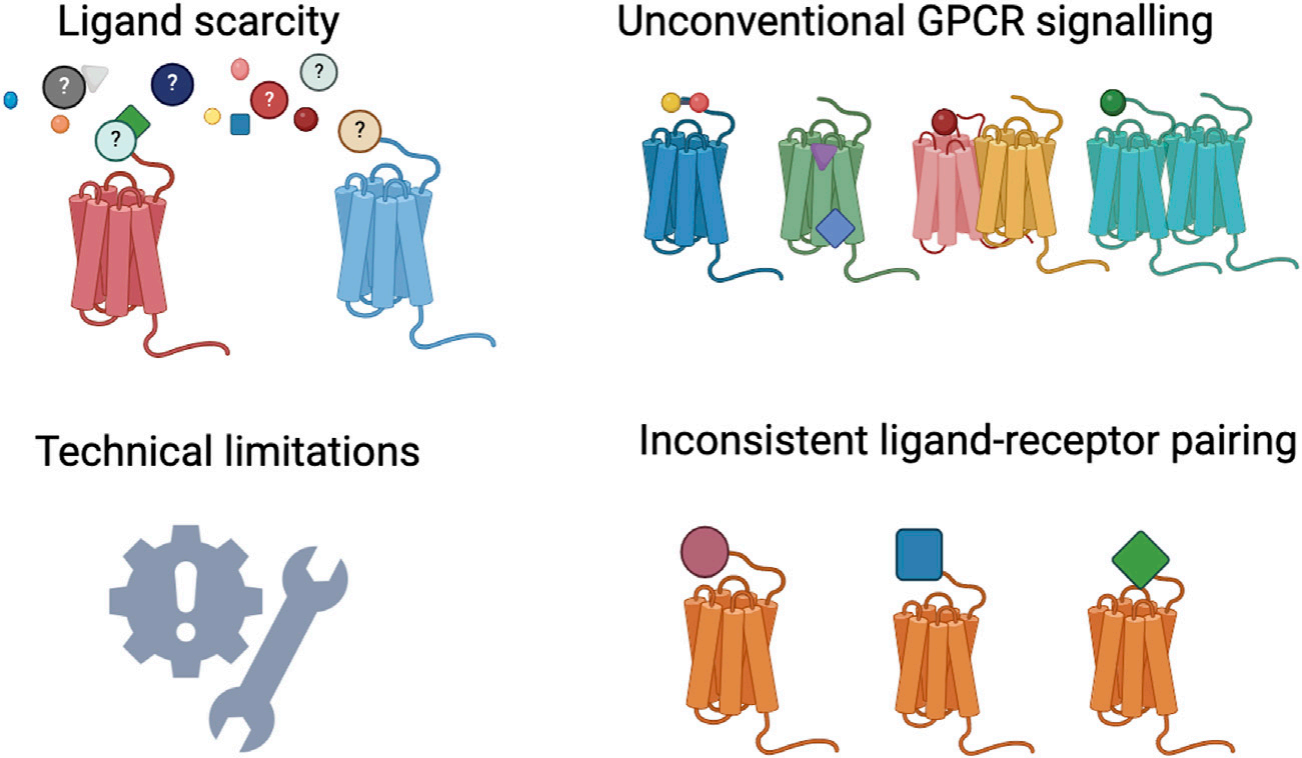
The main challenges to orphan GPCR deorphanization. Created with BioRender.com.

GPCRs signal primarily via guanine nucleotide-binding proteins or G proteins, which function as molecular switches for cellular signal transmission as illustrated in Figure 4. The main challenges associated with homing orphan GPCRs are illustrated in Figure 5. They also mediate G protein-independent pathways through GPCR kinases (GRKs), β-arrestins, regulators of G protein signaling (RGS), receptor activity-modifying proteins (RAMPs), and proteins PDZ motif-containing proteins. Such interactions modulate receptor activity or initiate other signaling pathways (Foster et al., 2015). In inducing numerous intracellular processes, GPCRs modulate second messenger levels, including intracellular Ca^2+,^ cAMP and cAMP response element binding (CREB), inositol triphosphate (IP3), and diacylglycerol (DAG) (Downes and Gautam, 1999). GPCR signaling is terminated through desensitization/receptor internalization which is mediated by GRK-induced phosphorylation of the C-terminal tail, resulting in G protein dissociation (Ferguson, 2001), and reinforcing receptor-β arrestin complex stability which precludes further rounds of receptor induction (Kühn et al., 1984; Lohse et al., 1992; Wilden, 1995; Krupnick et al., 1997). As per the classical paradigm, the internalized receptor is either recycled for subsequent receptor activation or directed to endosomes for lysosomal degradation. The recent notion that GPCRs continue to maintain G protein function post internalization led to a paradigm shift and further increases the multifaceted nature of GPCR signaling (Sutkeviciute and Vilardaga, 2020), with studies reporting unconventional GPCR signaling in intracellular enclosures such as early endosomes, mitochondria, nucleus, Golgi, and endoplasmic reticulum (ER) (Jong et al., 2018a; Jong et al., 2018b; Calebiro and Godbole, 2018; Eichel and Von Zastrow, 2018; Hanyaloglu, 2018; Lobingier and Von Zastrow, 2019; Retamal et al., 2019; Plouffe et al., 2020).

Besides the orthosteric region recognized by the majority of native ligands, GPCRs carry other binding regions which ensure allosteric modulation of receptor function (Hauser et al., 2017). Moreover, there are a few GPCRs in which ligands act mainly at the minor pocket between TM1 and 3, and TM7, as observed in GPR52 (a class A orphan), proteinase-activated (PAR1 and PAR2), glucagon (GLP-1) receptor, chemokine (CCR2, CCR6, CXCR2, and CXCR4), succinate (SUCNR1), leukotriene (BLT1), and prostanoid (EP3, EP4, and thromboxane A2) receptors (Ballante et al., 2021). Furthermore, presently unpaired orphan GPCRs may exhibit ligand-independent activities as some receptors mediate trafficking, or serve as ligand sinks (Wise et al., 2004). Such ligand-independent roles can be credited to constitutive activity and receptor dimerization with other protein partners. This applies to “true” orphans that remain to be paired and ‘conditional’ orphans that mimic orphans in the absence of a native ligand, as exemplified by the adopted ghrelin receptor (Ahmad et al., 2015). Aiming to elucidate the activation mechanism of GPR88 and to provide a model for structure-based drug design for neuropsychiatric disorders, Chen et al. (2022) recently uncovered a distinct activation mechanism for GPR88 with a water-mediated polar network and a set of defining structure features. The group also reported an electron density in the extracellular orthosteric site of orphan GPR88 that may signify a putative endogenous agonist.

#### 3.4.1 Constitutive and self-activating GPCRs

The homologous receptors GPR26 and GPR78 exhibit constitutive activity (Jones et al., 2007), similar to GPR3, GPR6, and GPR12, a subfamily that constitutively stimulates the adenylyl cyclase cascade (Mehlmann et al., 2004; Ledent et al., 2005; Tanaka et al., 2007). Additionally, some virus-induced orphan receptors such as the Epstein Barr virus-induced receptor 2 (Rosenkilde et al., 2005) and the human cytomegalovirus-encoded UL33 orphan GPCR (Waldhoer et al., 2002; Vischer et al., 2006) also feature similar basal activity (Levoye and Jockers, 2008). Such phenomenon has been previously related to the absence of the conserved DRY motif found in class A GPCRs (Wess, 1998) as observed in GPR20, GPR141, and GPR151. A mutation in this motif typically results in constitutive activity as shown by several studies (Rasmussen et al., 1999; Alewijnse et al., 2000; Scheer et al., 2000). In contrast, a mutation within the DRY motif fails to constitutively activate the α2-adrenergic receptor but rather enhances ligand affinity (Chung et al., 2002). Another possibility could be due to allosteric binding as exemplified by GPR119 which accommodates an allosteric lipid agonist (Xu et al., 2022). As observed for orphan GPR3, the extent of constitutive activity is influenced by receptor expression level and the regulatory proteins involved in signal termination, namely, β-arrestin 2 and GRK2 (Lowther et al., 2013). Toyooka’s group attributed the constitutive activity of some orphan receptors to the presence of a possible N-terminal ligand, as basal activity ceased following the truncation of the GPR16 N-terminal tail (Toyooka et al., 2009). Likewise, the melanocortin MC4 receptor exhibits N-terminus-dependent constitutive activity in addition to ligand-based activation (Ersoy et al., 2012). Lin et al. attribute the basal activity of GPR20 to an unusually coiled N-terminal helix cap on its transmembrane domain. They also report an orthosteric cavity which could be explored for deorphaization efforts considering that GPR20 is a potential biological target for gastrointestinal tumors (Lin et al., 2023). Alternatively, a seemingly constitutive state could also be due to a strongly bound native ligand (Laschet et al., 2018), as illustrated by GPR40, whose ligand cavity is occupied by native fatty acid ligands (Stoddart et al., 2007). Tethered/bound agonists have been reported for four orphan adhesion GPCRs: GPR64, GPR114, GPR126 and GPR133 (Araç et al., 2012) which all display constitutive functions. Such possibilities should be considered in inverse agonist screening efforts. On the contrary, the presence of inverse agonists may also conceal the ligand-binding region (Levoye and Jockers, 2008).

Interestingly, the constitutive activity of GPR52 which is implicated in Huntington’s disease is based on its self-activation (Lin et al., 2020) resulting from the presence of a ligand-like motif in ECL2 filling the orthosteric cavity, though the receptor also has a ligand-binding side pocket (Lin et al., 2020). GPR17 also exhibits a similar phenomenon (Ye et al., 2022), and so does type 2 diabetes-associated GPR21, the only homolog of GPR52 sharing 70% sequence identity (Wong et al., 2023). GPR62 (Muroi et al., 2017) and most recently GPR142 (Yasuda et al., 2023) have also been reported to exhibit constitutive activity.

In an effort to study nucleotide-sensitive associations among 48 understudied orphan GPCRs and five G proteins, Lu et al. (2021) performed bioluminescence resonance energy transfer (BRET) and concluded that the G protein association of constitutively active GPCRs can be characterized via the detection of receptor-G protein interactions in the absence of guanine nucleotides. This method could prove valuable for probing the G protein association of the remaining orphan GPCRs.

#### 3.4.2 Function of orphan GPCRs in dimeric complexes

Although GPCRs were originally regarded as single-unit proteins, they are now widely acknowledged to form dimeric and/or oligomeric complexes with other GPCRs that are distinct from their monomers with respect to physiological and pharmacological outcomes. Within such complexes, they modify ligand binding, receptor trafficking, interactions with intracellular scaffold proteins, and signaling cascades (Drakopoulos et al., 2022), in addition to mediating allosteric regulation (Ahmad et al., 2015). In another possibility, conditional heteromerization of related GPCRs can result in novel functionality through allostery, as depicted by the melatonin MT1 receptor with its fellow orphan GPR50; which negatively inhibits melatonin-mediated signaling (Levoye et al., 2006; Ahmad et al., 2015). An online application for predicting GPCR-GPCR interaction pairs is offered by the GGIP web server (Nemoto et al., 2022). This tool could potentially be used to predict the possibility of a given orphan receptor engaging in dimeric complexes with other GPCR members.

Orphan GPCRs usually heterodimerize with characterized GPCRs belonging to the same subfamily as illustrated by the ligand-bound GABAB1 and orphan GABAB2 which lacks a GABA-binding domain and instead serves in signal transmission (Kniazeff et al., 2002). Similarly, GPR179 engages in heterodimerization with the metabotropic glutamate receptor (mGlu6) (Orlandi et al., 2013). Another example is the heterodimerization of the MAS-related receptor MrgD with the GPCR MrgE, which augments signaling and inhibits receptor internalization of MrgD (Milasta et al., 2006). Conversely, the MT1 melatonin receptor is negatively modulated by the orphan GPR50 in a heterodimer complex (Levoye et al., 2006). Orphan GPCRs also engage in heteromeric complexes with non-GPCR membrane proteins, enzymes, transporters, cellular proteins (Ahmad et al., 2015) and ion channels (Levoye and Jockers, 2008). For instance, the orphan GPR37 which is linked to Parkinson’s disease has been reported to interact with the dopamine transporter DAT (Ahmad et al., 2015). Of note, most of these complexes occur constitutively without ligand activation (Levoye and Jockers, 2008). Furthermore, the activation of certain orphan GPCRs may require accessory proteins (Chung et al., 2008), as shown for the calcitonin receptor, which requires RAMPs to activate its signaling pathway (McLatchie et al., 1998; Hay et al., 2006).

Altogether, such heterogeneity of GPCR complexes poses a great challenge in terms of establishing the activity and evaluating the subsequent effects on downstream intracellular signaling of a putative ligand for a given orphan receptor. Firstly, the interacting protein in a heterodimer complex can influence ligand activity, exhibiting unique pharmacological and signaling properties distinct from those of the individual monomers. Secondly, there is growing evidence suggesting that heterodimer composition affects agonist function (Barnes, 2006), making the functional consequence of heterodimerization even more unpredictable. Thirdly, functional crosstalk between different pathways at the level of receptors, G proteins, second messengers or effectors signaling events (Cordeaux and Hill, 2002) poses an even further challenge. However, this phenomenon can also occur independent of oligomerization as observed in Class C GPCRs such as metabotropic glutamate (mGlu) receptors, calcium and GABAB receptors (Prezeau et al., 2010). Additionally, ligand activity at a given GPCR may be dependent on tissue type (Insel et al., 2012) owing to tissue-specific expression. This adds another layer of complexity in elucidating orphan GPCR signaling. Ultimately, the challenge lies in relating the specific role of a given orphan GPCR within complex signaling networks.

#### 3.4.3 Promiscuous and non-selective GPCRs

GPCR subfamilies often bind one or more similar ligands (Civelli et al., 2006) as seen with three opioid receptors all of which engage opioid peptides (Meng et al., 1998; Reinscheid et al., 1998). Catecholamine receptors also bear structural relatedness as do their ligands (Lanau et al., 1997). Even further, GPRC6A is activated by a set of basic L-α-amino acids, with an affinity for basic amino acid residues (Wellendorph et al., 2005). Contrarily, despite evidence that adrenaline and noradrenalin stimulate the dopamine D4 receptor, the adrenergic and dopaminergic systems are categorized as two separate receptor sub-families (Lanau et al., 1997). Likewise, the Mas-related GPCRs have been coupled to various structurally distinct transmitters (Zylka et al., 2003). Interestingly, certain GPCRs, namely, GPR105 are activated by unexpected neurotransmitters such as UDP-glucose (Chambers et al., 2000). Similarly, two closely related former orphans, GPR91 (Wittenberger et al., 2001) and GPR99 (Wittenberger et al., 2002), respond to citric acid cycle intermediaries succinate and α-ketoglutarate, respectively (He et al., 2004). Certain GPCRs, such as GPR119 and GPR132 also show promiscuity for a variety of bacterial and human fatty acid amides (FAAs) (Chang et al., 2021).

In summary, the lack of endogenous ligands and the knowledge gap in orphan GPCR receptor pharmacology impede advancements in orphan GPCR deorphanization. Due to technical limitations related to assay sensitivity, specificity, and throughput; the classical functional assays that have driven the field thus far prove inadequate for realizing the deorphanization of the remaining orphan GPCRs. Moreover, the structural plasticity and functional heterogeneity of GPCRs make the development of a standardized deorphanization assay a rather impossible task. This has been well-acknowledged in recent studies which take more integrative approaches to cover the multiple facets of GPCR signaling.

## 4 Discussion

The function of GPCRs goes beyond conventional GPCR signaling mechanisms and extends to other functions involving participation in multiprotein complexes and constitutive activity. Thus future deorphanization efforts need to take into account such atypical and at times peculiar nature of GPCR signaling to fulfill the unmet quest for the identification of endogenous ligands of the most therapeutically significant receptors. Additionally, future homing of orphan GPCRs may not necessarily take a one-size-fits-all approach but should be more comprehensive in accounting for numerous possibilities to cover the full range of GPCR signaling. Moving forward, integrative approaches toward orphan GPCR deorphanization efforts as demonstrated by Foster et al. (2019) would prove more fruitful. Though the scarcity of 3D crystallized structures is not ideal, it does not disqualify accurate ligand prediction, as exemplified by recent machine learning-powered technologies which enable the deorphanization of orphan GPCRs despite no structural input. Nevertheless, further developments toward deep learning models for GPCR loop modelling and refinement could greatly improve the accuracy of predicted structures.

An important aspect to consider during experimental validation of predicted pairings is the possible PTMs of GPCRs. Mass spectrometry-based proteomics and high-resolution crystal structures can be particularly useful in mapping PTM sites. Since drug specificity is vital, it can prove clinically beneficial to account for differential GPCR modulation in varying cellular environments. In this regard, the concept of biased agonism—toward either the G protein or β-arrestin pathway—has seen recently rising interest. The pathway of choice can be influenced by the given cellular environment, which is contingent on the group of GPCR-associating proteins, localization, or even trafficking depending on the cell type (Ritter and Hall, 2009). Using high-content imaging in combination with parallel assays can identify compounds that would otherwise go unnoticed using a single platform. Apart from unveiling biased agonists, this integrative approach could be particularly relevant in GPCR drug discovery. Though docking screens report biased signaling of numerous discovered ligands, the structural basis of such biased agonism remains to be elucidated (Ballante et al., 2021). To computationally capture biased agonism, more effort should be directed toward machine learning models that predict signatures of biased agonists. In an attempt to explore scaffolds and pharmacophores that confer bias to either G protein or β-arrestin, a recent study (Sanchez et al., 2021) took this approach by considering GPCR ligands from the BiasDB database that display biased signaling. While a higher content of secondary and aromatic amines seems to be indicative of β-arrestin bias, this is however not conclusive.

Future efforts should take into account other factors such as cellular environment and receptor conformation. At present, organoids and 3D cell cultures which offer a great substitute for standard cultures in cell-based assays remain an untapped resource in the field. Such advanced models provide a better physiologically suited environment and could prove more suitable for exploring orphan GPCR pharmacology. This could possibly remedy the challenges related to the low levels of receptor expression in *in vitro* studies. Considering the challenges in terms of tissue-specificity and transient expression; advances in single-cell RNA sequencing can offer insights into functional heterogeneity in different tissues under various states.

Moving forward, the correction of irreproducible ligand-receptor pairs listed by Laschet et al. (2018) also warrants future attention. This may include testing for constitutive activity, predicting possible GPCR dimerization partners, and the use of multiple orthogonal assays to cover the full spectrum of GPCR signaling.

Interestingly, orphan receptors GPR33 and GPR42 were previously considered pseudogenes but have been recently demonstrated to be functional (Puhl et al., 2015; Morri et al., 2018), respectively. As per the IUPHAR, GPR79, TAAR2, TAAR3, and TAAR4P remain characterized as pseudogenes. Hence, probing the possible functionality of such claimed pseudogenes may add to the functional GPCR repertoire and ultimately unravel new ligand pairings. Since the gut microbiome has been implicated in metabolic, cardiovascular, neurodegenerative, and gastrointestinal diseases (Chen et al., 2019), the screening of microbial products against orphan GPCRs may reveal novel orphan GPCR-ligand pairings. Furthermore, a compilation of endogenous ligand libraries will facilitate the *in silico* pairing of orphan receptors.

In light of the therapeutic significance of GPCRs, further efforts are required to design effective drugs for the already characterized members. The crystallization of identified GPCRs with either native or synthetic agonists in different conformations will aid in drug discovery efforts. On that note, there is rising interest in drugs acting at several GPCRs implicated in a specific disease (Anighoro et al., 2014). As multitarget activity is a key feature of many antipsychotic medications, polypharmacology may result in synergistic therapeutic effects (Roth et al., 2004). In this regard, orphan receptors GPR88 and GPR124 (Calderón-Zamora et al., 2017) with potential implications in the development of hypertension offer a context for polypharmacological drug discovery. Like most GPCR ligands, the majority of orphan lipids are expressed in nervous tissue. Thus, investigating the possibility of interaction between orphan lipids and orphan GPCRs merits further effort. As reported by (Bradshaw et al., 2009), an attempt to deorphanize orphan GPCRs along with the over 70 endogenous lipids with a basic N-acyl amide structure presents an untapped opportunity for a more comprehensive picture of cellular signaling and an endeavour “to find them all a home.” Considering their therapeutic relevance, the search for native orphan GPCR ligands continues.
