# Supplementary material for: Orphan G protein-coupled receptors: the ongoing search for a home
Source: Front Pharmacol. 2024 Feb 29;15:1349097. doi: 10.3389/fphar.2024.1349097 (PMC10941346; doi:10.3389/fphar.2024.1349097)
Supplement: Supplementary file 1 [file Table1.pdf]

# Orphan G protein-coupled receptors: The ongoing search for a home

Amie Jobe[1], Ranjit Vijayan[1,2,3,*]

[1] Department of Biology, College of Science, United Arab Emirates University, Al Ain, United Arab Emirates
[2] The Big Data Analytics Center, United Arab Emirates University, Al Ain, United Arab Emirate
[3] Zayed bin Sultan Center for Health Sciences, United Arab Emirates University, Al Ain, United Arab Emirates

* Corresponding author: ranjit.v@uaeu.ac.ae

## Supplementary Material

**Appendix SA1.** List of orphan GPCRs as per the IUPHAR/BPS Guide to Pharmacology database made publicly available under the Creative Commons Attribution-ShareAlike 4.0 International license (https://creativecommons.org/licenses/by-sa/4.0/). The proposed ligands/agonists, if any, are shown in grey. Created with BioRender.com.

## Rhodopsin (Class A)

GPR3
sphingosine 1-phosphate

GPR4
protons

GPR42 (probable pseudogene)

GPR6
sphingosine 1-phosphate

GPR12
sphingosine 1-phosphate

GPR15
ATP, LTC4, LTD4, LTE4, UDP, UDP-galactose UDP-glucose, cysteinyl-leukotrienes (CysLTs), uracil nucleotides

GPR17
adropin

GPR19
adropin

GPR20 (constitutive activity)

GPR21

GPR22

GPR25

GPR26

GPR27

GPR31
12S-HETE

GPR32
resolvin D1, LXA4

GPR33 (pseudogene in most individuals)

GPR34
lysophosphatidylserine

GPR35
kynurenic acid, 2-oleoyl-LPA

GPR37
prosaptide, prosaposin

GPR37L1
prosaptide, prosaposin

GPR39
obestatin, $Zn^{2+}$

GPR45

GPR50 (heterodimerizes constitutively with $MT_1$ and $MT_2$ melatonin receptors)

GPR52 (constitutive activity)

GPR61 (constitutive activity)

GPR62

GPR63
dihydrosphingosine 1-phosphate
dioleoylphosphatidic acid
sphingosine 1-phosphate

GPR65
protons

GPR68
protons

GPR75
CCL5

GPR78 (constitutive activity)

GPR79 (pseudogene)

GPR82

GPR83
neuropeptide PEN

GPR84
Medium-chain-length fatty acids

GPR85

GPR87
LPA

GPR88

GPR101

GPR132
9-hydroxyoctadecadienoic acid
(lyso)phospholipid mediators, protons

GPR 135
L-phenylalanine, L-tryptophan

GPR139
L-phenylalanine, L-tryptophan

GPR141

GPR142
L-phenylalanine, L-tryptophan

GPR146
proinsulin C-peptide

GPR148

GPR149

GPR150

GPR151 (possibly structurally similar to galanin)

GPR152

GPR153

GPR160

GPR161

GPR162

GPR171
peptide BigLEN

GPR173

GPR174
lysophosphatidylserine

GPR176

GPR182
adrenomedullin (in rat)

GPR183
7α,27-dihydroxycholesterol
7β, 27-dihydroxycholesterol
7β, 25-dihydroxycholesterol
7α,25-dihydroxycholesterol
27-hydroxycholesterol
25-hydroxycholesterol
7α-hydroxycholesterol
7β-hydroxycholesterol
Oxysterols

LGR4
R-spondin-1, R-spondin-2
R-spondin-3, R-spondin-4

LGR5
R-spondin-1, R-spondin-2
R-spondin-3, R-spondin-4

LGR6
R-spondin-1, R-spondin-2
R-spondin-3, R-spondin-4

MAS1

MAS1L

MRGPRD
β-alanine

MRGPRE

MRGPRF
angiotensin metabolites

MRGPRG

MRGPRX1
bovine adrenal medulla - peptide 8-22

MRGPRX2
PAMP-20

MRGPRX3

MRGPRX4

P2RY8

P2RY10
LPA
sphingosine 1-phosphate

TAAR2
β-phenylethylamine tryptamine

TAAR3P (probable pseudogene)
isoamylamine

TAAR4P (pseudogene)

TAAR5

TAAR6

TAAR8

TAAR9

## Adhesion (Class B2)

ADGRG1
TYFAVLM derived peptides

ADGRG2
TSFGVLLDLSRTSLPP derived peptides

ADGRG3

ADGRG4

ADGRG5
TYFAVLMQLSGDPVPAEL derived peptides

ADGRG6
THFGVLMDLPRSASQL derived-peptides
progesterone and 17-hydroxyprogesterone

ADGRG7

ADGRL1
lasso D

ADGRL2
α-latrotoxin

ADGRL3
FLRT3 (in rat)

ADGRL4

ADGRV1

ADGRA1

ADGRA2
glycosaminoglycans

ADGRA3
GA-binding protein gamma (GABPγ)
glutaminase interacting protein

ADGRB1
phosphatidylserine

ADGRB2

ADGRB3

CELSR1
Vangl-2, Frizzled-6, LRRK

CELSR2

CELSR3

ADGRD1
PLXDC2
THLTNFAILMQVV derived-peptides

ADGRD2

ADGRE1

ADGRE2

ADGRE3

ADGRE4P (pseudogene)

ADGRE5

ADGRF1
synaptamide
TSFSILMSPFVPSTIFPVVKWIT derived peptides

ADGRF2P (probable pseudogene)

ADGRF3

ADGRF44
TSFSILMSPDSPD derived peptides

ADGRF5
TSFSILMSPDSPD derived peptides

## Glutamate (Class C)

GPR156

GPR158

GPR179

GPRC5A

GPRC5B

GPRC5C

GPRC5D

GPRC6 receptor
glycine, L-alanine
L-arginine, L-citrulline
L-glutamine, L-lysine
L-ornithine, L-serine

## Opsins

OPN1LW

OPN1MW

OPN1SW

Rhodopsin

OPN3 (Probable sensory receptor)

OPN4

OPN5

## Taste 1 receptors

TAS1R1
glutamate
L-glutamic acid
guanosine monophosphate
inosine monophosphate

TAS1R2
sucrose, saccharin

TAS1R3
L-glutamic acid
sucrose, saccharin

## Taste 2 receptors

TAS2R1
cohumulone, L-Phe-Phe-Phe, dextromethorphan
L-Trp-Trp-Trp

TAS2R3
chloroquine

TAS2R4
L-Trp-Trp-Trp, azithromycin, stevioside, colchicine

TAS2R5
epigallocatechin-3-gallate, Procyanidin C2
1,10-Phenanthroline

TAS2R7
grandinin, malvidin-3-glucoside, cromoglicic acid
aluminum sulfate, magnesium sulfate

TAS2R8
oleuropein, andrographolide, chloramphenicol, parthenolide

TAS2R9
ofloxacin, pirenzepine, procainamide

TAS2R10
bergapten, cucurbitacin B, strychnine, denatonium, haloperidol

TAS2R13
diphenidol, denatonium

TAS2R14
flufenamic acid, aristolochic acid, nobiletin, luteolin, santonin
datiscetin, parthenolide, (-)-α-thujone, picrotoxinin
N-octanoyl-L-homoserine lactone, phloretin, resveratrol, tributyrin
eriodictyol chalcone, (±)-Equol, silibinin, (+/-)-Eriodictyol
genistein, homoeriodictyol, coumestro, vanillin, lupulone, quinine
L-Trp-Trp-Trp

TAS2R16
4-Nitrophenyl-β-D-mannopyranoside, Phenyl-β-D-glucopyranoside
salicin, beta-gentiobiose, D-(-)-Amygdalin, sinigrin

TAS2R19

TAS2R20
ritanserin, methoxsalen, cromoglicic acid, tobramycin, vanillin
diphenidol

TAS2R30
denatonium, absinthin, amarogentin

TAS2R30
denatonium, absinthin, amarogentin

TAS2R31
aristolochic acid, saccharin, acesulfame, famotidine

TAS2R38
propylthiouracil, phenylthiocarbamide, goitrin
methimazole, sinigrin

TAS2R39
theaflavin-3'-O-gallate, theaflavin, luteolin, epicatechin
gallate, naringenin, scutellarein, phloretin, datiscetin
genistein, (±)-Equol, epigallocatechin
(-)-Epicatechin, vanillin, L-Trp-Trp-Trp

TAS2R40
cohumulone, quinine, dapsone

TAS2R41
chloramphenicol

TAS2R42

TAS2R43
aristolochic acid, lactucopicrin, aloin, Cyclolinopeptide 1-
Mso,3-Met-CL6, bengalensol, grosheimin, amarogentin
saccharin, acesulfame

TAS2R45

TAS2R46
lactucopicrin, strychnine, grosheimin, absinthin,
bengalensol, andrographolide, amarogentin, picrotoxinin
denatonium, colchicine, L-Trp-Trp-Trp

TAS2R50
andrographolide, amarogentin

TAS2R60

## Other 7TM receptors

GPR107
neuronostatin

GPR137

GPR143
levodopa

GPR157
